# The crystal structure of (*RS*)-7-chloro-2-(2,5-di­meth­oxy­phen­yl)-2,3-di­hydro­quinazolin-4(1*H*)-one: two hydrogen bonds generate an elegant three-dimensional framework structure

**DOI:** 10.1107/S2056989019007023

**Published:** 2019-05-21

**Authors:** Kereyagalahally H. Narasimhamurthy, Chandra, Belakavadi K. Sagar, Kanchugarakoppal S. Rangappa, Hemmige S. Yathirajan, Christopher Glidewell

**Affiliations:** a Department of Studies in Organic Chemistry, University of Mysore, Manasagangotri, Mysuru-570 006, India; b Department of Physics, National Institute of Engineering, Mysore-570 008, India; c Department of Studies in Chemistry, University of Mysore, Manasagangotri, Mysuru-570 006, India; d School of Chemistry, University of St Andrews, St Andrews, Fife KY16 9ST, UK

**Keywords:** heterocyclic compounds, reduced quinazolinones, crystal structure, mol­ecular conformation, hydrogen bonding, supra­molecular assembly

## Abstract

In the title compound, C_61_H_15_ClN_2_O_3_, the heterocyclic ring adopts an envelope conformation, folded across the N⋯N line, with the 2,5-di­meth­oxy­phenyl unit occupying a quasi-axial site. There are two N—H⋯O hydrogen bonds in the structure: one hydrogen bond links mol­ecules related by a 4_1_ screw axis to form a *C*(6) chain, and the other links inversion-related pairs of mol­ecules to form an *R*_2_^2^(8) ring. The ring motif links all of the chains into a continuous three-dimensional framework structure. Comparisons are made with the structures of some related compounds.

## Abstract 2

Two independent N—H⋯O hydrogen bonds link all of the mol­ecules into a continuous three-dimensional framework structure. The quinazoline ring adopts an envelope conformation with the 2,5-di­meth­oxy­phenyl­unit occupying a quasi-axial site.

## Chemical context   

**Figure 1:**
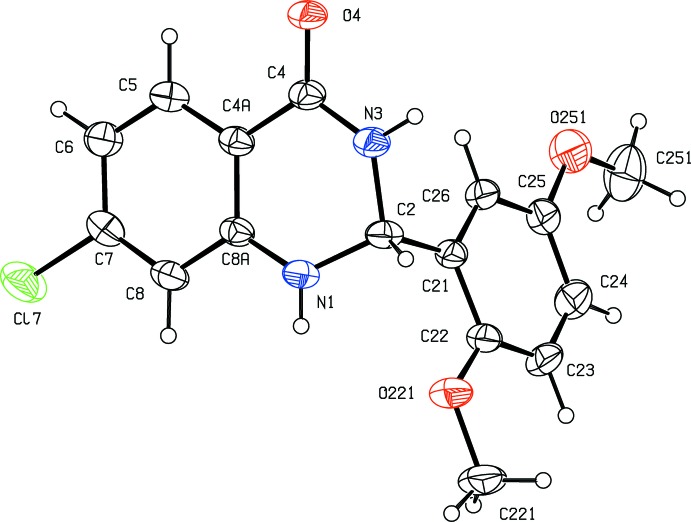
The mol­ecular structure of compound (I) showing the atom-labelling scheme. Displacement ellipsoids are drawn at the 30% probability level.

Quinazoline-4-one and its derivatives constitute an important class of fused heterocycles, which are found in more than two hundred naturally occurring alkaloids. In addition, 2,3-di­hydro­quinazolin-4(1*H*)-one is a privileged scaffold in drug design (Badolato *et al.*, 2018 ▸). Despite this, rather few structures have been published for compounds containing this heterocyclic nucleus (see Section 4 below), and with these considerations in mind, we now report the mol­ecular and supra­molecular structure of (*RS*)-7-chloro-2-(2,5-di­meth­oxy­phen­yl)-2,3-di­hydro­quinazolin-4(1*H*)-one (I) (Fig. 1 ▸). The compound was prepared using a recently published (Narasimhamurthy *et al.*, 2014 ▸) one-step process, which employs a base-promoted cyclization reaction between a (di­bromo­meth­yl)arene, here 2-(di­bromo­meth­yl)-1,4-di­meth­oxy­benz­ene, and a 2-amino­benzamide, here 2-amino-4-chloro­benzamide, which after a straightforward purification step gives the product (I) in 79% yield.
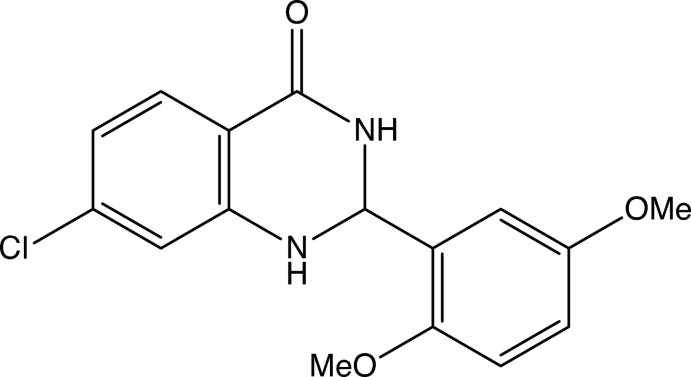


## Structural commentary   

The mol­ecule of compound (I) contains a stereogenic centre at atom C2, and the reference mol­ecule was selected as one having the *R* configuration at this atom: the centrosymmetric space group confirms that compound (I) has crystallized as a racemic mixture. The heterocyclic ring in compound (I) adopts a conformation close to the envelope form, in which this ring is folded across the line N1⋯N3 (Fig. 1 ▸). The ring-puckering parameters, calculated for the atom sequence (N1,C2,N3,C4,C4*A*,C8*A*) in the *R*-enanti­omer are *Q* = 0.258 (2) Å, θ = 121.8 (4)° and φ = 219.3 (6)°. For the ideal envelope form, the puckering angles take the values θ = 54.7° (equivalent to 125.3°) and φ = (60*k*)°, where *k* represents an integer (Boeyens, 1978 ▸). The r.m.s. deviation of the atoms N1, N3, C4, C4*A*, C8*A* from their mean plane is only 0.035 Å, with a maximum deviation of 0.0403 (11) Å for atom N3. However, atom C2 is displaced from this plane by 0.355 (3) Å. The 2,5-di­meth­oxy­phenyl substituent occupies the quasi-axial site at atom C2. Within this unit, the two meth­oxy C atoms are almost coplanar with the aryl ring: the deviations from the mean plane of this ring are 0.020 (5) Å for atom C221 and 0.101 (5) Å for atom C251. Associated with this planarity, the two exocyclic C—C—O angles at atoms C22 and C25 are significantly different, by 11.9° at C22 and by 8.2° at atoms C25, as previously observed in planar or near-planar alk­oxy­arenes (Seip & Seip, 1973 ▸; Ferguson *et al.*, 1996 ▸).

## Supra­molecular features   

**Figure 2:**
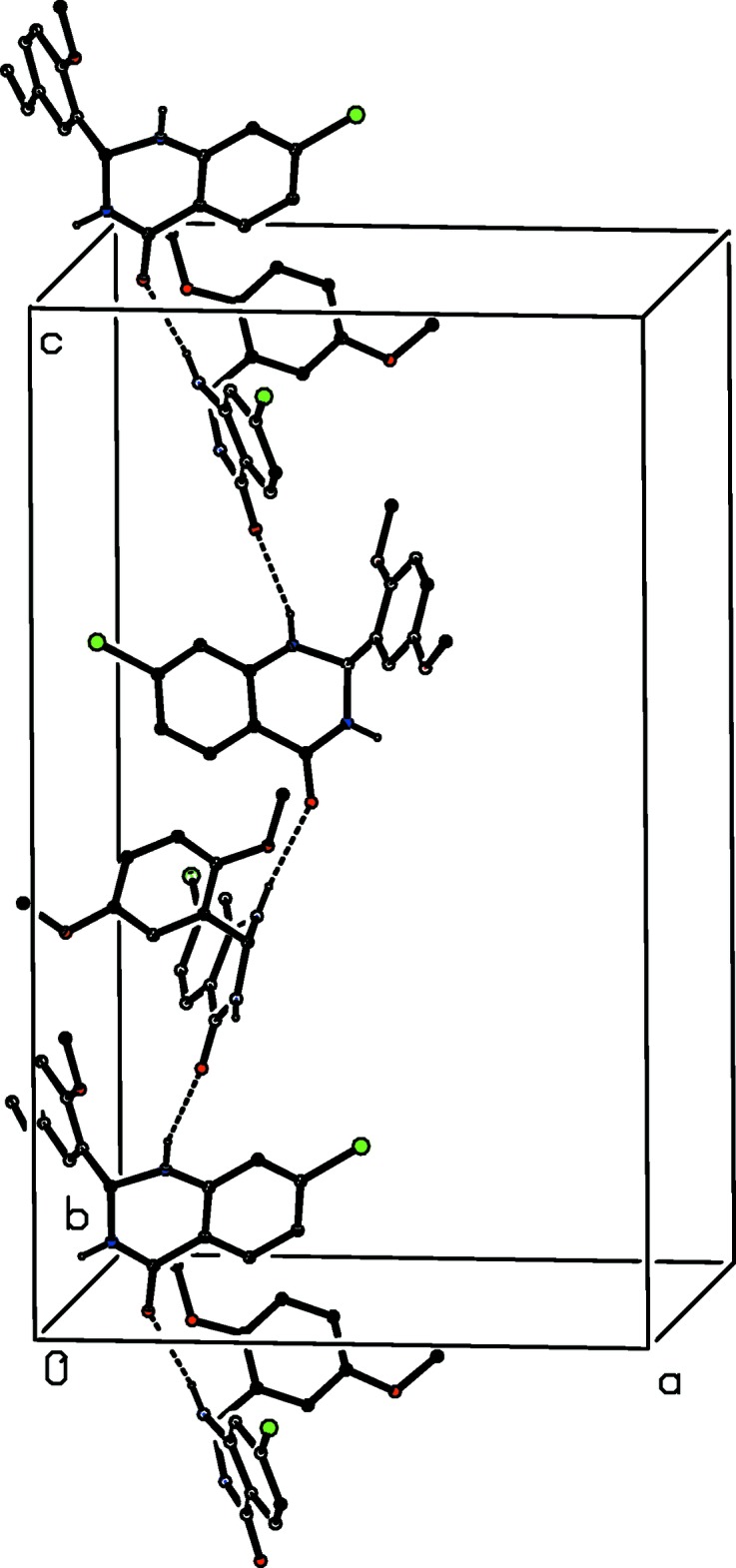
Part of the crystal structure of compound (I) showing the formation of a hydrogen-bonded *C*(6) chain running parallel to [001]. Hydrogen bonds are drawn as dashed lines and, for the sake of clarity, the H atoms bonded to C atoms have been omitted.

**Figure 3:**
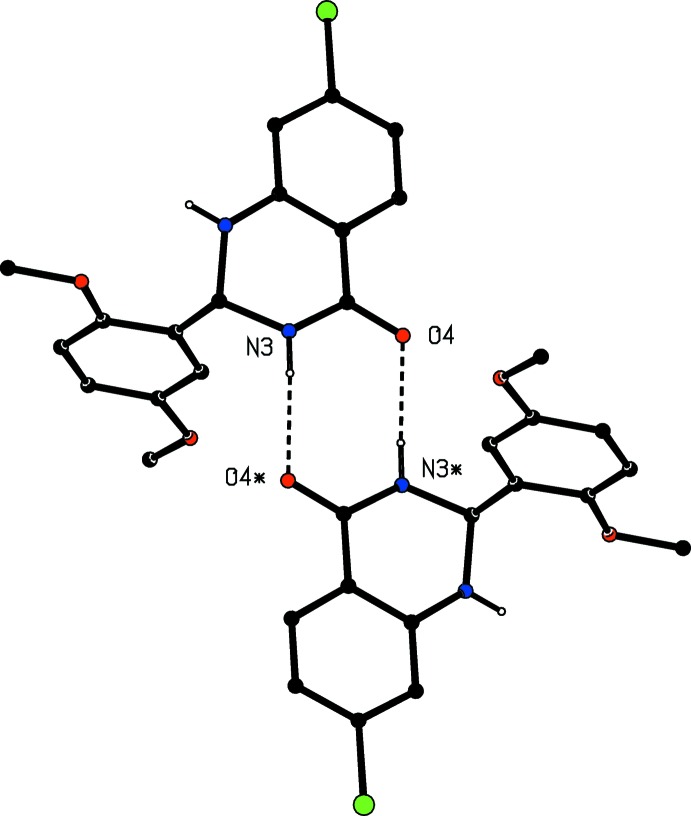
Part of the crystal structure of compound (I) showing the formation of a cyclic hydrogen-bonded dimer. Hydrogen bonds are drawn as dashed lines and, for the sake of clarity, the unit-cell outline and the H atoms bonded to C atoms have been omitted. The atoms marked with an asterisk (*) are at the symmetry position (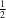 − *x*, 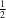 − *y*, 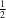 − *z*).

**Figure 4:**
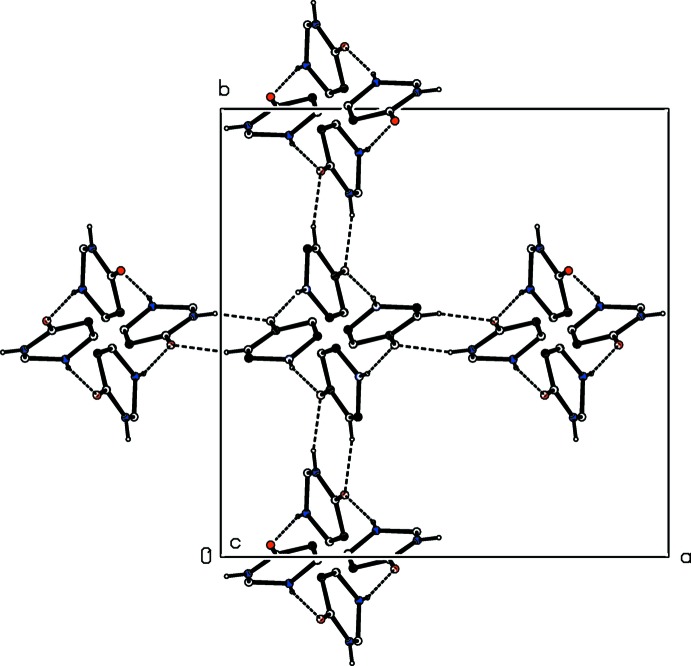
A projection along [001] of part of the crystal structure of compound (I) showing the linking of the *C*(6) chains by the 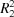
(8) rings. Hydrogen bonds are drawn as dashed lines and, for the sake of clarity, only the heterocyclic ring, along with its hydrogen-bond acceptors and donors, is shown for each mol­ecule.

The structure of compound (I) contains just two N—H⋯O hydrogen bonds (Table 1 ▸) but these are sufficient to link all of the mol­ecules into a three-dimensional framework structure, whose formation is readily analysed in terms of the actions of the two individual hydrogen bonds. The hydrogen bond having atom N1 as the donor links mol­ecules related by the 4_1_ screw axis along (0.25, 0.5, *z*) into a *C*(6) chain (Etter, 1990 ▸; Etter *et al.*, 1990 ▸; Bernstein *et al.*, 1995 ▸) running parallel to the [001] direction (Fig. 2 ▸). Four chains of this type pass through each unit cell. The hydrogen bond having atom N3 as the donor links inversion-related pairs of mol­ecules to form a cyclic dimer characterized by an 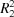
(8) motif (Fig. 3 ▸). This inter­action directly links the *C*(6) chain around the 4_1_ screw axis (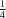, 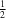, *z*) with four similar chains around the screw axes along (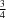, 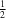, *z*), (−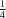, 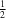, *z*), (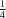, 0, *z*) and (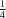, 1, *z*) (Fig. 4 ▸). Propagation of these hydrogen bonds by the space-group symmetry operations links all of the *C*(6) chains, so linking all of the mol­ecules into a very elegant three-dimensional structure generated by only two hydrogen bonds.

## Database survey   

**Figure 5:**
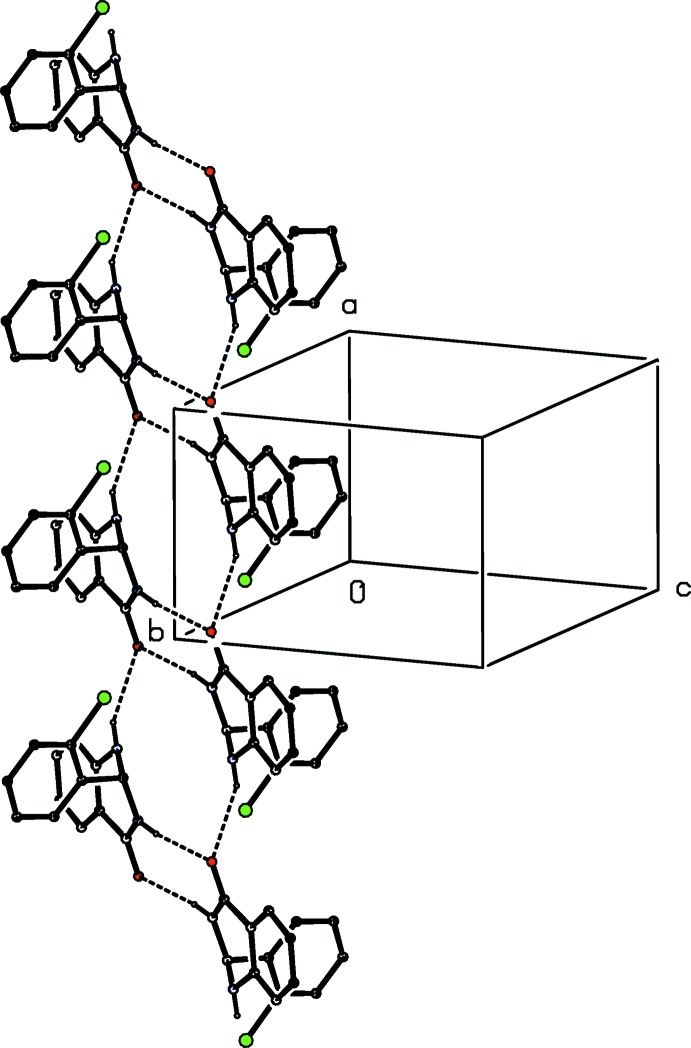
Part of the crystal structure of (*RS*)-2-(2-chloro­phen­yl)-2,3-di­hydro­quinazolin-4(1*H*)-one showing the formation of a hydrogen-bonded chain of edge-fused rings along [100]. The published atomic coordinates (Li & Feng, 2009 ▸) have been used. Hydrogen bonds are drawn as dashed lines and, for the sake of clarity, the H atoms bonded to C atoms have been omitted.

It is of inter­est briefly to compare the mol­ecular and supra­molecular structure of (I) reported here with those of some related structures. In (*RS*)-2-(2-chloro­phen­yl)-2,3-di­hydro­quinazolin-4(1*H*)-one (Li & Feng, 2009 ▸), the heterocyclic ring has a screw–boat conformation, as opposed to the envelope form in (I). As in (I), the structure contains two N—H⋯O hydrogen bonds, and these were described in the original report as generating a polymer along *b*, but without further specification. However, examination of the published atomic coordinates shows clearly that the mol­ecules are linked into a chain of centrosymmetric, edge-fused rings running parallel to the [100] direction, in which 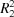
(8) rings centred at (*n*, 1, 0) alternate with 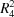
(12) rings centred at (*n* + 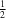, 1, 0), where *n* represents an integer in each case (Fig. 5 ▸).

In 5-chloro-3-hy­droxy-2,2-dimethyl-2,3-di­hydro­quinazolin-4(1*H*)-one (Vembu *et al.*, 2006 ▸), the heterocyclic ring again adopts the screw–boat conformation, and a combination of N—H⋯O and O—H⋯O hydrogen bonds links the mol­ecules into complex sheets, within which rings of *S*(5), 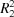
(4) and 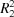
(10) types can be identified. There is no carbonyl group in (*RS*)-2-methyl-4-phenyl-3,4-di­hydro­quinazoline, and here mol­ecules which are related by a 3_1_ screw axis are linked by an N-*–*H⋯N hydrogen bond to form a *C*(5) chain (Valkonen *et al.*, 2011 ▸).

Finally, we note the structures of a number of 2,3-di­hydro­quinazolin-4(1*H*)-ones in which there is a substituent at atom N3 (Butcher *et al.*, 2007 ▸; Toze *et al.*, 2018 ▸; Zaytsev *et al.*, 2018 ▸). In each of these examples, the mol­ecules are linked by a single N—H⋯O hydrogen bond to form a *C*(6) chain. However, when the substituent at atom N3 is an aryl­methyl­amino group, the heterocyclic ring adopts a screw–boat conformation (Butcher *et al.*, 2007 ▸), but in five examples where this substituent is either a benzyl group or a furan­ylmethyl unit, the heterocyclic ring adopts an envelope conformation, folded across the N⋯N line (Toze *et al.*, 2018 ▸; Zaytsev *et al.* 2018 ▸).

## Synthesis and crystallization   

A sample of compound (I) was prepared using a recently published general procedure (Narasimhamurthy *et al.*, 2014 ▸). Potassium *tert*-butoxide (3.3 mmol) was added to a suspension of 2-(di­bromo­meth­yl)-1,4-di­meth­oxy­benzene (3.3 mmol) and 2-amino-4-chloro­benzamide (3.5 mmol) in a pyridine-di­methyl­formamide mixture (3:1, *v*/*v*). The resulting mixture was heated at 313 K for 4 h, with TLC monitoring. When the reaction was judged to be complete, an excess of water was added, followed by extraction with ethyl acetate (2 × 20 ml). The combined organic extract was washed with brine and then dried over anhydrous sodium sulfate. The solvent was removed under reduced pressure and the crude product was purified by column chromatography using silica gel mesh 60–120, with 30% ethyl acetate in hexane as eluent, to give the product (I) in 79% yield. Crystals suitable for single crystal X-ray diffraction were grown by slow evaporation, at ambient temperature and in the presence of air, of a solution in di­methyl­sulfoxide: m.p. 481–483 K.

## Refinement   

Crystal data, data collection and structure refinement details are given in Table 2 ▸. In the setting of space group *I*4_1_/*a*, No. 88, employed here the origin is located at a centre of inversion. All H atoms were located in difference maps. The H atoms bonded to C atoms were then treated as riding atoms in geometrically idealized position with C—H 0.93 Å (aromatic), 0.96 Å (CH_3_) or 0.98 Å (aliphatic C—H), and with *U*_iso_(H) = *kU*_eq_(C), where *k* = 1.5 for the methyl groups, which were permitted to rotate but not to tilt, and 1.2 for all other H atoms bonded to C atoms. For the H atoms bonded to N atoms, the atomic coordinates were refined with *U*_iso_(H) = 1.2*U*_eq_(N), giving the N—H distances shown in Table 1 ▸.

## Supplementary Material

Crystal structure: contains datablock(s) global, I. DOI: 10.1107/S2056989019007023/zl2756sup1.cif

Structure factors: contains datablock(s) I. DOI: 10.1107/S2056989019007023/zl2756Isup2.hkl

Supporting information file. DOI: 10.1107/S2056989019007023/zl2756Isup3.cml

CCDC reference: 1875494

Additional supporting information:  crystallographic information; 3D view; checkCIF report

## Figures and Tables

**Table 1:** Hydrogen-bond geometry (Å, °)

*D*—H⋯*A*	*D*—H	H⋯*A*	*D*⋯*A*	*D*—H⋯*A*
N1—H1⋯O4^i^	0.80 (3)	2.39 (3)	3.161 (3)	162 (2)
N3—H3⋯O4^ii^	0.83 (3)	2.04 (3)	2.854 (3)	166 (2)

Symmetry codes: (i) 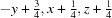; (ii) 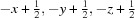.

**Table 2:** Experimental details

Crystal data	
Chemical formula	C_16_H_15_ClN_2_O_3_
*M*_r_	318.75
Crystal system, space group	Tetragonal, *I*4_1_/*a*
Temperature (K)	296
*a*, *c* (Å)	15.314 (7), 25.736 (12)
*V* (Å^3^)	6036 (6)
*Z*	16
Radiation type	Mo *K*α
μ (mm^−1^)	0.27
Crystal size (mm)	0.26 × 0.22 × 0.18

Data collection	
Diffractometer	Bruker APEXII CCD
Absorption correction	Multi-scan (*SADABS*; Bruker, 2015 ▸)
*T*_min_, *T*_max_	0.913, 0.953
No. of measured, independent and observed [*I* > 2σ(*I*)] reflections	42994, 3149, 1848
*R*_int_	0.072
(sin θ/λ)_max_ (Å^−1^)	0.629

Refinement	
*R*[*F* ^2^ > 2σ(*F* ^2^)], *wR*(*F* ^2^), *S*	0.049, 0.128, 1.04
No. of reflections	3149
No. of parameters	207
H-atom treatment	H atoms treated by a mixture of independent and constrained refinement
Δρ_max_, Δρ_min_ (e Å^−3^)	0.17, −0.27

Computer programs: *APEX2* and *SAINT* (Bruker, 2015 ▸), *SHELXS97* (Sheldrick, 2008 ▸), *SHELXL2014* (Sheldrick, 2015 ▸) and *PLATON* (Spek, 2009 ▸).

## supplementary crystallographic information

### Crystal data

**Table:**

C_16_H_15_ClN_2_O_3_	*D*_x_ = 1.403 Mg m^−^^3^
*M**_r_* = 318.75	Mo *K*α radiation, λ = 0.71073 Å
Tetragonal, *I*4_1_/*a*	Cell parameters from 3465 reflections
*a* = 15.314 (7) Å	θ = 1.6–27.6°
*c* = 25.736 (12) Å	µ = 0.27 mm^−^^1^
*V* = 6036 (6) Å^3^	*T* = 296 K
*Z* = 16	Block, colourless
*F*(000) = 2656	0.26 × 0.22 × 0.18 mm

### Data collection

**Table:**

Bruker APEXII CCD diffractometer	3149 independent reflections
Radiation source: fine focus sealed tube	1848 reflections with *I* > 2σ(*I*)
Graphite monochromator	*R*_int_ = 0.072
Detector resolution: 0.3333 pixels mm^-1^	θ_max_ = 26.6°, θ_min_ = 1.6°
φ and ω scans	*h* = −19→19
Absorption correction: multi-scan (SADABS; Bruker, 2015)	*k* = −19→19
*T*_min_ = 0.913, *T*_max_ = 0.953	*l* = −32→26
42994 measured reflections	

### Refinement

**Table:**

Refinement on *F*^2^	Primary atom site location: difference Fourier map
Least-squares matrix: full	Hydrogen site location: mixed
*R*[*F*^2^ > 2σ(*F*^2^)] = 0.049	H atoms treated by a mixture of independent and constrained refinement
*wR*(*F*^2^) = 0.128	*w* = 1/[σ^2^(*F*_o_^2^) + (0.0505*P*)^2^ + 3.5139*P*] where *P* = (*F*_o_^2^ + 2*F*_c_^2^)/3
*S* = 1.03	(Δ/σ)_max_ < 0.001
3149 reflections	Δρ_max_ = 0.17 e Å^−^^3^
207 parameters	Δρ_min_ = −0.27 e Å^−^^3^
0 restraints	

### Special details

**Table:**

Geometry. All esds (except the esd in the dihedral angle between two l.s. planes) are estimated using the full covariance matrix. The cell esds are taken into account individually in the estimation of esds in distances, angles and torsion angles; correlations between esds in cell parameters are only used when they are defined by crystal symmetry. An approximate (isotropic) treatment of cell esds is used for estimating esds involving l.s. planes.

### Fractional atomic coordinates and isotropic or equivalent isotropic displacement parameters (Å^2^)

**Table:**

	*x*	*y*	*z*	*U*_iso_*/*U*_eq_	
N1	0.30909 (14)	0.40264 (14)	0.37953 (7)	0.0531 (6)	
H1	0.3275 (17)	0.4092 (16)	0.4083 (10)	0.064*	
C2	0.30616 (16)	0.31258 (15)	0.36210 (8)	0.0482 (6)	
H2	0.3646	0.2879	0.3670	0.058*	
N3	0.28752 (14)	0.31121 (14)	0.30684 (7)	0.0506 (5)	
H3	0.2932 (16)	0.2628 (17)	0.2929 (9)	0.061*	
C4	0.24435 (15)	0.37295 (16)	0.28060 (8)	0.0467 (6)	
O4	0.22338 (12)	0.36121 (11)	0.23471 (6)	0.0603 (5)	
C4A	0.22505 (14)	0.45429 (15)	0.30792 (8)	0.0434 (5)	
C5	0.17657 (16)	0.51985 (16)	0.28452 (9)	0.0521 (6)	
H5	0.1566	0.5118	0.2507	0.062*	
C6	0.15743 (16)	0.59599 (17)	0.30984 (9)	0.0569 (7)	
H6	0.1250	0.6397	0.2939	0.068*	
C7	0.18794 (16)	0.60558 (16)	0.35987 (9)	0.0556 (6)	
Cl7	0.16054 (6)	0.70075 (5)	0.39245 (3)	0.0873 (3)	
C8	0.23775 (16)	0.54326 (16)	0.38428 (9)	0.0522 (6)	
H8	0.2583	0.5527	0.4178	0.063*	
C8A	0.25726 (14)	0.46570 (15)	0.35832 (8)	0.0424 (5)	
C21	0.24297 (15)	0.25723 (14)	0.39324 (8)	0.0432 (5)	
C22	0.26659 (17)	0.23605 (16)	0.44405 (8)	0.0522 (6)	
C23	0.2110 (2)	0.18716 (17)	0.47413 (9)	0.0678 (8)	
H23	0.2271	0.1735	0.5080	0.081*	
C24	0.1321 (2)	0.15794 (18)	0.45543 (10)	0.0682 (8)	
H24	0.0951	0.1257	0.4767	0.082*	
C25	0.10822 (18)	0.17630 (16)	0.40582 (9)	0.0559 (7)	
C26	0.16454 (16)	0.22579 (15)	0.37492 (8)	0.0488 (6)	
H26	0.1485	0.2379	0.3408	0.059*	
O221	0.34622 (12)	0.26875 (12)	0.45915 (6)	0.0718 (6)	
C221	0.3739 (2)	0.2493 (2)	0.51083 (11)	0.0918 (11)	
H22A	0.3823	0.1874	0.5143	0.138*	
H22B	0.3302	0.2684	0.5350	0.138*	
H22C	0.4279	0.2789	0.5179	0.138*	
O251	0.03307 (13)	0.14750 (13)	0.38268 (8)	0.0767 (6)	
C251	−0.0300 (2)	0.1052 (2)	0.41418 (14)	0.0939 (11)	
H25A	−0.0488	0.1442	0.4412	0.141*	
H25B	−0.0048	0.0539	0.4294	0.141*	
H25C	−0.0793	0.0889	0.3932	0.141*	

### Atomic displacement parameters (Å^2^)

**Table:**

	*U*^11^	*U*^22^	*U*^33^	*U*^12^	*U*^13^	*U*^23^
N1	0.0655 (14)	0.0565 (13)	0.0373 (10)	−0.0097 (11)	−0.0105 (10)	−0.0042 (10)
C2	0.0520 (14)	0.0582 (15)	0.0343 (12)	0.0045 (12)	−0.0054 (10)	−0.0063 (10)
N3	0.0645 (14)	0.0545 (13)	0.0328 (10)	0.0040 (11)	0.0019 (9)	−0.0061 (9)
C4	0.0508 (14)	0.0574 (15)	0.0321 (11)	−0.0045 (12)	0.0049 (10)	−0.0021 (11)
O4	0.0872 (13)	0.0620 (11)	0.0316 (8)	0.0061 (9)	−0.0056 (8)	−0.0085 (7)
C4A	0.0427 (13)	0.0524 (14)	0.0351 (11)	−0.0070 (11)	0.0026 (10)	−0.0054 (10)
C5	0.0539 (15)	0.0618 (16)	0.0404 (12)	−0.0005 (13)	−0.0050 (11)	−0.0082 (11)
C6	0.0585 (16)	0.0595 (17)	0.0528 (14)	0.0039 (13)	−0.0050 (12)	−0.0054 (12)
C7	0.0568 (16)	0.0551 (16)	0.0548 (14)	−0.0041 (13)	0.0012 (12)	−0.0154 (12)
Cl7	0.1085 (7)	0.0736 (5)	0.0800 (5)	0.0165 (4)	−0.0147 (4)	−0.0338 (4)
C8	0.0580 (16)	0.0593 (16)	0.0395 (12)	−0.0130 (13)	−0.0033 (11)	−0.0104 (11)
C8A	0.0404 (13)	0.0508 (14)	0.0359 (11)	−0.0119 (11)	0.0002 (10)	−0.0033 (10)
C21	0.0560 (15)	0.0410 (13)	0.0326 (11)	0.0068 (11)	−0.0025 (10)	−0.0045 (9)
C22	0.0692 (17)	0.0490 (15)	0.0384 (12)	0.0063 (13)	−0.0121 (12)	−0.0073 (11)
C23	0.109 (2)	0.0588 (17)	0.0354 (13)	0.0081 (17)	−0.0032 (14)	0.0095 (12)
C24	0.088 (2)	0.0621 (18)	0.0540 (16)	−0.0059 (16)	0.0068 (15)	0.0091 (13)
C25	0.0707 (18)	0.0458 (14)	0.0514 (14)	0.0010 (13)	0.0006 (13)	−0.0025 (11)
C26	0.0612 (16)	0.0484 (14)	0.0369 (11)	0.0042 (12)	−0.0039 (11)	0.0006 (10)
O221	0.0866 (14)	0.0768 (13)	0.0521 (11)	0.0029 (11)	−0.0292 (10)	−0.0026 (9)
C221	0.124 (3)	0.091 (2)	0.0607 (17)	0.024 (2)	−0.0496 (18)	−0.0082 (16)
O251	0.0707 (13)	0.0784 (14)	0.0810 (13)	−0.0176 (11)	−0.0024 (11)	0.0074 (11)
C251	0.072 (2)	0.082 (2)	0.128 (3)	−0.0106 (17)	0.012 (2)	0.026 (2)

### Geometric parameters (Å, º)

**Table:**

N1—C8A	1.364 (3)	C21—C26	1.377 (3)
N1—C2	1.451 (3)	C21—C22	1.395 (3)
N1—H1	0.80 (3)	C22—C23	1.373 (4)
C2—N3	1.451 (3)	C22—O221	1.374 (3)
C2—C21	1.516 (3)	C23—C24	1.376 (4)
C2—H2	0.9800	C23—H23	0.9300
N3—C4	1.337 (3)	C24—C25	1.357 (3)
N3—H3	0.83 (2)	C24—H24	0.9300
C4—O4	1.237 (3)	C25—O251	1.369 (3)
C4—C4A	1.461 (3)	C25—C26	1.397 (3)
C4A—C5	1.386 (3)	C26—H26	0.9300
C4A—C8A	1.399 (3)	O221—C221	1.427 (3)
C5—C6	1.368 (3)	C221—H22A	0.9600
C5—H5	0.9300	C221—H22B	0.9600
C6—C7	1.377 (3)	C221—H22C	0.9600
C6—H6	0.9300	O251—C251	1.418 (3)
C7—C8	1.374 (3)	C251—H25A	0.9600
C7—Cl7	1.733 (3)	C251—H25B	0.9600
C8—C8A	1.395 (3)	C251—H25C	0.9600
C8—H8	0.9300		
			
C8A—N1—C2	122.09 (19)	C26—C21—C22	117.8 (2)
C8A—N1—H1	119.2 (19)	C26—C21—C2	124.84 (19)
C2—N1—H1	114.6 (19)	C22—C21—C2	117.4 (2)
N3—C2—N1	108.84 (19)	C23—C22—O221	126.1 (2)
N3—C2—C21	112.62 (19)	C23—C22—C21	119.6 (2)
N1—C2—C21	112.82 (18)	O221—C22—C21	114.2 (2)
N3—C2—H2	107.4	C22—C23—C24	121.7 (2)
N1—C2—H2	107.4	C22—C23—H23	119.2
C21—C2—H2	107.4	C24—C23—H23	119.2
C4—N3—C2	125.6 (2)	C25—C24—C23	119.9 (3)
C4—N3—H3	117.8 (17)	C25—C24—H24	120.1
C2—N3—H3	114.6 (17)	C23—C24—H24	120.1
O4—C4—N3	120.5 (2)	C24—C25—O251	124.7 (2)
O4—C4—C4A	122.1 (2)	C24—C25—C26	118.8 (3)
N3—C4—C4A	117.39 (19)	O251—C25—C26	116.5 (2)
C5—C4A—C8A	120.1 (2)	C21—C26—C25	122.2 (2)
C5—C4A—C4	121.12 (19)	C21—C26—H26	118.9
C8A—C4A—C4	118.8 (2)	C25—C26—H26	118.9
C6—C5—C4A	121.7 (2)	C22—O221—C221	116.8 (2)
C6—C5—H5	119.2	O221—C221—H22A	109.5
C4A—C5—H5	119.2	O221—C221—H22B	109.5
C5—C6—C7	117.6 (2)	H22A—C221—H22B	109.5
C5—C6—H6	121.2	O221—C221—H22C	109.5
C7—C6—H6	121.2	H22A—C221—H22C	109.5
C8—C7—C6	122.8 (2)	H22B—C221—H22C	109.5
C8—C7—Cl7	119.83 (19)	C25—O251—C251	118.1 (2)
C6—C7—Cl7	117.4 (2)	O251—C251—H25A	109.5
C7—C8—C8A	119.4 (2)	O251—C251—H25B	109.5
C7—C8—H8	120.3	H25A—C251—H25B	109.5
C8A—C8—H8	120.3	O251—C251—H25C	109.5
N1—C8A—C8	122.4 (2)	H25A—C251—H25C	109.5
N1—C8A—C4A	119.2 (2)	H25B—C251—H25C	109.5
C8—C8A—C4A	118.4 (2)		
			
C8A—N1—C2—N3	33.6 (3)	C5—C4A—C8A—C8	1.3 (3)
C8A—N1—C2—C21	−92.2 (2)	C4—C4A—C8A—C8	−179.4 (2)
N1—C2—N3—C4	−26.5 (3)	N3—C2—C21—C26	−14.9 (3)
C21—C2—N3—C4	99.4 (3)	N1—C2—C21—C26	108.8 (2)
C2—N3—C4—O4	−171.9 (2)	N3—C2—C21—C22	164.5 (2)
C2—N3—C4—C4A	9.3 (3)	N1—C2—C21—C22	−71.8 (3)
O4—C4—C4A—C5	4.7 (3)	C26—C21—C22—C23	−1.5 (3)
N3—C4—C4A—C5	−176.6 (2)	C2—C21—C22—C23	179.0 (2)
O4—C4—C4A—C8A	−174.6 (2)	C26—C21—C22—O221	179.1 (2)
N3—C4—C4A—C8A	4.1 (3)	C2—C21—C22—O221	−0.4 (3)
C8A—C4A—C5—C6	−1.3 (4)	O221—C22—C23—C24	179.5 (2)
C4—C4A—C5—C6	179.4 (2)	C21—C22—C23—C24	0.2 (4)
C4A—C5—C6—C7	−0.1 (4)	C22—C23—C24—C25	1.0 (4)
C5—C6—C7—C8	1.5 (4)	C23—C24—C25—O251	177.2 (2)
C5—C6—C7—Cl7	−178.02 (19)	C23—C24—C25—C26	−0.8 (4)
C6—C7—C8—C8A	−1.5 (4)	C22—C21—C26—C25	1.8 (3)
Cl7—C7—C8—C8A	178.07 (18)	C2—C21—C26—C25	−178.8 (2)
C2—N1—C8A—C8	158.6 (2)	C24—C25—C26—C21	−0.6 (4)
C2—N1—C8A—C4A	−24.0 (3)	O251—C25—C26—C21	−178.8 (2)
C7—C8—C8A—N1	177.4 (2)	C23—C22—O221—C221	0.6 (4)
C7—C8—C8A—C4A	0.0 (3)	C21—C22—O221—C221	179.9 (2)
C5—C4A—C8A—N1	−176.2 (2)	C24—C25—O251—C251	8.7 (4)
C4—C4A—C8A—N1	3.1 (3)	C26—C25—O251—C251	−173.4 (2)

### Hydrogen-bond geometry (Å, º)

**Table:**

*D*—H···*A*	*D*—H	H···*A*	*D*···*A*	*D*—H···*A*
N1—H1···O4^i^	0.80 (3)	2.39 (3)	3.161 (3)	162 (2)
N3—H3···O4^ii^	0.83 (3)	2.04 (3)	2.854 (3)	166 (2)

Symmetry codes: (i) −*y*+3/4, *x*+1/4, *z*+1/4; (ii) −*x*+1/2, −*y*+1/2, −*z*+1/2.
